# Supplementary material for: Enteroviruses Manipulate the Unfolded Protein Response through Multifaceted Deregulation of the Ire1-Xbp1 Pathway
Source: Viruses. 2022 Nov 10;14(11):2486. doi: 10.3390/v14112486 (PMC9699254; doi:10.3390/v14112486)
Supplement: Supplementary file 1 [file viruses-14-02486-s001.zip › viruses-1899862-supplementary.pdf]

Article

Enteroviruses Manipulate the Unfolded Protein Response through Multifaceted deregulation of the Ire1-Xbp1 Pathway

Anna Shishova, Ilya Dyugay, Ksenia Fominykh, Victoria Baryshnikova, Alena Dereventsova, Yuriy Turchenko, Anna A. Slavokhotova, Yury Ivin, Sergey E. Dmitriev, and Anatoly Gmyl

Supplementary materials

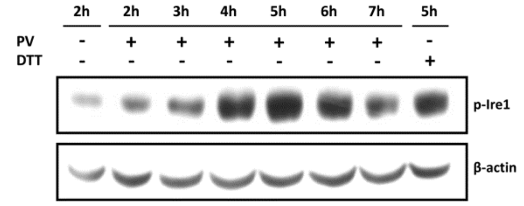

Figure S1. Ire1 phosphorylation in poliovirus-infected cells (another representative result in addition to that shown in Figure 1, with a more comprehensive time-course). Phosphorylated Ire1 and total β-actin levels in HeLa cells infected with PV type I Mahoney were revealed by Western blot analysis using anti-p-Ire1 and anti-ACTB antibodies, respectively.

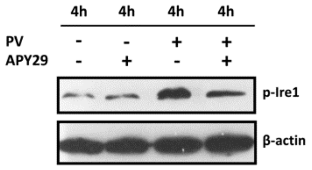

Figure S2. Ire1 phosphorylation at the middle stage of enterovirus infection is dependent on its kinase activity. HeLa cells was infected with PV, then the Ire1 autophosphorylation inhibitor APY29 (Cayman Chemical, #22913, up to 180 μM) was added into the medium, as indicated. Cells were harvested at 4 hpi. Phosphorylated Ire1 and total β-actin levels were analyzed by Western blot analysis.

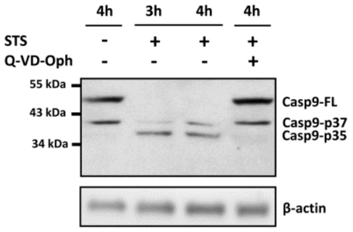

Figure S3. Q-VD-Oph inhibits staurosporine-induced caspase-9 cleavage. HeLa cells were treated with 1 μM staurosporine in the presence or absence of 20 μM Q-VD-Oph. After the time indicated, cells were lysed and analyzed by Western blotting using anti-caspase 9 antibodies. p35 and p37 represent caspase-9 cleavage products.

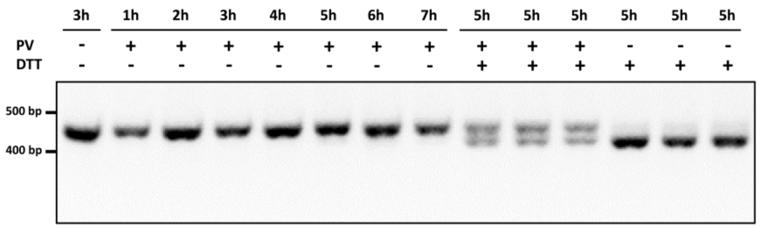

Figure S4. The time-course of Xbp1 mRNA splicing in PV-infected cells. HeLa cells were infected with PV and harvested at different time points. When indicated, 10 mM DTT was added 2 h before the harvesting (at 3 hpi). Then, RNA was extracted and RT-PCR was performed with an Xbp1-specific primer pair producing fragments of either 442 or 416 bp (corresponding to the unspliced or spliced Xbp1 mRNAs, respectively). The right part of the gel represents three independent replicates of DTT-treated (PV-infected or mock-infected) cells. Note that the amounts of two PCR products should not be compared to each other, as they have different lengths.

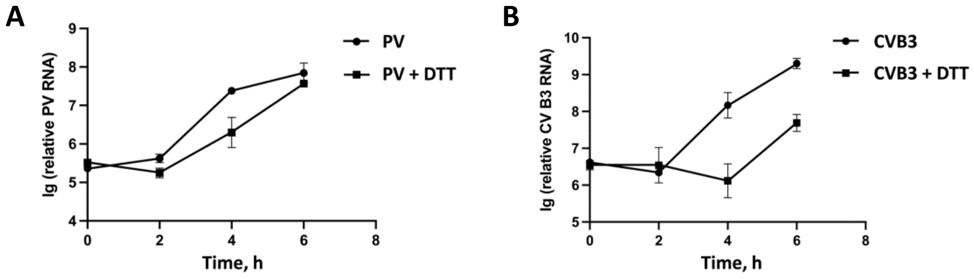

Figure S5. DTT treatment does not significantly affect the course of enterovirus infection in HeLa cells, as confirmed by RT-qPCR of the PV **(A)** and CVB3 **(B)** genomic RNAs. DTT was added to the growth medium immediately after infection.

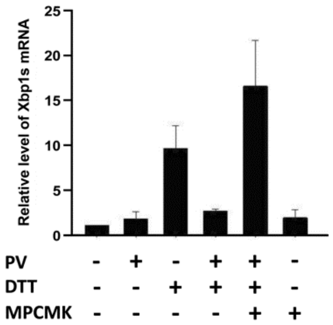

Figure S6. MPCMK, a viral 2A protease inhibitor, abrogates the effects of PV infection on Xbp1 mRNA splicing. HeLa cells were infected with PV or mock-infected in the presence or absence of 650 μM MPCMK, as indicated. At 2 hpi, 10 mM DTT was added to the medium. 2 h later, total RNA was isolated and the relative level of the spliced Xbp1 mRNA was measured by RT-qPCR with primers specific to the Xbp1s mRNA isoform and to *RPL19* mRNA as a reference. The value for mock-infected cells was taken as 1.

Copyright: © 2022 by the authors. Licensee MDPI, Basel, Switzerland. This article is an open access article distributed under the terms and conditions of the Creative Commons Attribution (CC BY) license (https://creativecommons.org/licenses/by/4.0/).
